# Robotic Nephrectomy in a Patient With a Single Kidney: An Approach Ensuring Safety and Success

**DOI:** 10.7759/cureus.90824

**Published:** 2025-08-23

**Authors:** Soumi Pathak, Manoj Bhardwaj

**Affiliations:** 1 Anaesthesiology, Rajiv Gandhi Cancer Institute and Research Centre, New Delhi, IND

**Keywords:** chronic, fluid therapy, nephrectomy, renal dialysis, renal insufficiency

## Abstract

Perioperative anesthetic management for radical nephrectomy in a previous post-nephrectomy patient poses various challenges and requires thorough clinical and radiological evaluation. A comprehensive plan, including point-of-care intensive monitoring to prevent major fluid shifts, acidemia, and maintain electrolyte homeostasis, as well as robot-assisted surgery, is essential for achieving optimal surgical outcomes. In this case report, we describe the perioperative management of a 70-year-old male patient, a postoperative case of right radical nephrectomy, who was scheduled for left robotic nephrectomy. Maintaining a mean arterial pressure (MAP) above 65 mmHg, achieving an euvolemic state, and preventing acidosis, hyperkalemia, and hypernatremia were our primary targets; however, achieving these goals proved quite challenging. Serial arterial blood gas (ABG) analysis, ultrasound-guided B-line scoring, advanced cardiac monitoring, and an appropriate combination of anesthetic agents with minimal renal excretion and a short duration of action facilitated the successful management of the patient.

## Introduction

Renal cell carcinoma (RCC) is recognized as the most lethal of the urologic malignancies [1,2]. Approximately 20% to 30% of patients with RCC present with metastatic disease at the time of diagnosis, and more than 40% ultimately succumb to it. Robotic or laparoscopic partial nephrectomy for localized disease remains the mainstay of treatment. Radical nephrectomy is often recommended for large tumors (>4 cm). The incidence of recurrence or metastatic disease following radical nephrectomy ranges from approximately 20% to 40%, with contralateral kidney involvement occurring in less than 2% of cases [3,4]. When bilateral nephrectomy is required, renal replacement therapy becomes essential. The perioperative management of patients who are dependent on dialysis is complicated by disturbances in fluid and electrolyte homeostasis, as well as altered drug clearance. After ultrafiltrate hemodialysis, patients often have a hypovolemic blood compartment with lower oncotic pressure. Furthermore, as most anesthetic agents are myocardial depressants and have systemic vasodilatory activity, rapid succession of anesthesia with hemodialysis within seven hours is associated with an increased incidence of hypotension and related complications [5]. In this case report, we discuss the successful management of a 70-year-old male patient with a single kidney who was scheduled for robotic nephrectomy. Informed consent was obtained from the patient.

## Case presentation

**Figure 1:**
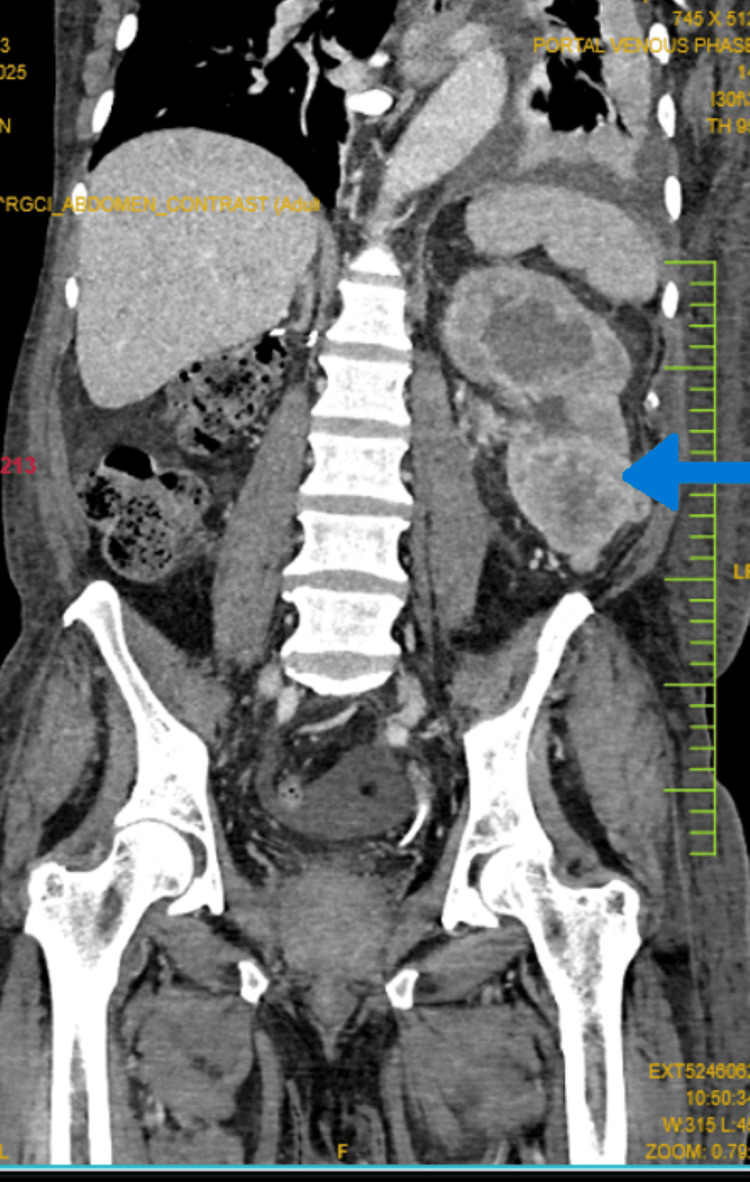
CT scan showing single large left kidney The left kidney appears diffusely bulky with a mildly enlarged exophytic growth at the lower pole (arrow). Post-right nephrectomy status is noted, with no definitive evidence of recurrent disease in the right renal fossa.

A 70-year-old male patient, a postoperative case of right radical nephrectomy, presented to our hospital with complaints of hematuria. CT scan revealed a 3.5 cm mass in his left kidney (Figure 1), which was infiltrating into the pelvic calyceal system (PCS) and ureter. Additionally, another lesion measuring 1.5 cm was found adjacent to the primary mass.

Due to the infiltration into the PCS, the patient was deemed unsuitable for focal therapy or nephron-sparing surgery. He commenced immunotherapy; however, he subsequently developed acute kidney injury (AKI) necessitating hemodialysis three times weekly. In light of disease progression and persistent hematuria, a left robotic nephrectomy was planned. Upon evaluation, the patient's laboratory investigations were as given in Table 1.

**Table 1:** Laboratory investigations #: decreased hemoglobin; *: increased creatinine; *#: increased serum potassium; ##: increased NT-proBNP NT-proBNP: N-terminal pro-B-type natriuretic peptide; APTT: activated partial thromboplastin time; PT: prothrombin time; INR: international normalized ratio

S.No.	Parameter	Patient values	Normal range
1	Hemoglobin	8.5 #	12.3-15.3 g/dL
2	Platelet count	186000	150000-400000
3	Urea	44	7-20 mg/dL
4	Creatinine	4.5 *	0.6-1.1 mg/L
5	Serum sodium	145 mmol/L	134-144 mmol/L
6	Serum potassium	5.7 mmol/L *#	3.5-4.5 mmol/L
7	NT-proBNP	1500 ##	<125 pg/mL
8	PT	12.7	11.7-13.1
9	INR	1.06	0.8-1.1
10	APTT	33.2	25.2-35.1

Additionally, the patient's glomerular filtration rate (GFR) was less than 30 mL/minute/1.73 m². Chest X-ray indicated bilateral pleural effusion, more pronounced on the left side. On auscultation, there was decreased breath sound on the left side of his chest, and there were no added sounds. His echocardiography (ECHO) showed an ejection fraction of 56%, and his ECG and stress ECHO were normal. He was hypertensive, diabetic, and had chronic kidney disease (CKD) grade 3. After optimisation of his comorbid conditions, he underwent hemodialysis the day before the surgery through a dialysis catheter (Certofix® Trio HF; B. Braun, Melsungen, Germany). Before surgery, an informative discussion was held with the patient and his attendant regarding the potential risks of the surgery and the long-term considerations associated with hemodialysis, including possible critical illness, intensive care unit (ICU) admission, and related perioperative morbidity and mortality. Following this thorough counseling and the acquisition of informed written consent, the patient was prepared to proceed with a left robotic nephrectomy.

**Figure 2:**
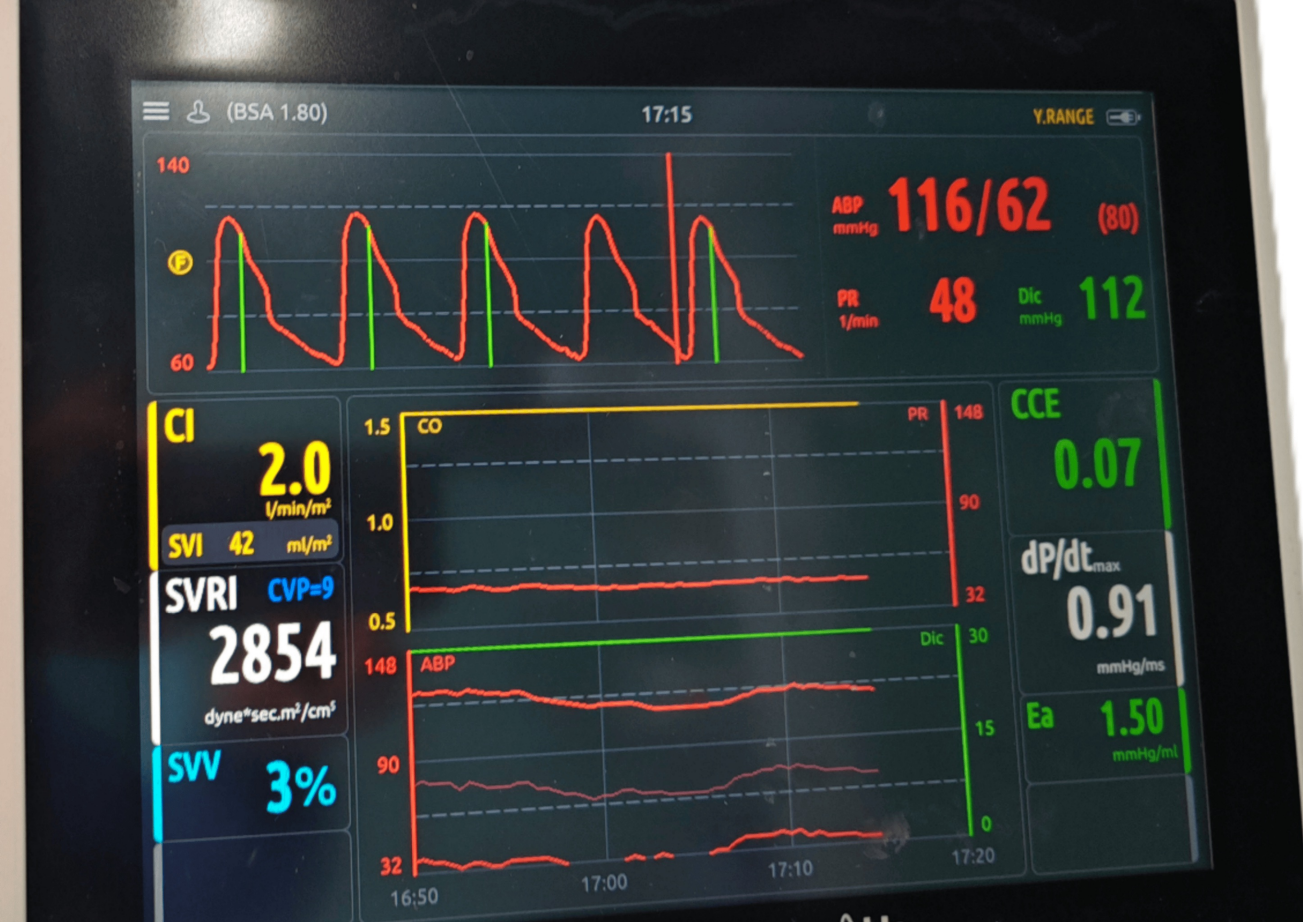
Cardiac output monitor results Cardiac output monitor showing SVV 3% (adequate fluid status), cardiac index 2.0 L/min/m^2^, dp/dt 0.9 mmHg/ms (adequate contractility of myocardium), Ea 1.50 mmHg/mL, and SVRI 2854 dyne·s·m^2^/cm^5^. SVV: Stroke volume variation; Ea: Elastance; SVRI: Systemic vascular resistance index; ABP: Arterial blood pressure; PR: Pulse rate; SVI: Stroke volume index; dp/dt: Rate of change of pressure within the left ventricle during the isovolumic contraction phase; CCE: Cardiac cycle efficiency; Dic: Dicrotic pressure

On the day of surgery, he was transferred to the OR after receiving ranitidine and granisetron. His baseline arterial blood gas (ABG) analysis was grossly normal with a pH of 7.4, PaO_2_ of 88 mmHg, PaCO_2_ of 35 mmHg, serum lactate of 1.8 mmol/L, and serum bicarbonate of 22 mEq/L. Standard monitors were then applied. A 16-G intravenous access was established on the right hand, and anesthesia was induced with the injection of fentanyl (2 µg/kg), propofol, and cisatracurium, and maintained using propofol, dexmedetomidine, cisatracurium infusion, sevoflurane, and a combination of air and oxygen, targeting a bispectral index (BIS) of 40-50. The infusion of cisatracurium was titrated based on neuromuscular monitoring. His right radial artery and left internal jugular vein were cannulated. Additionally, a cardiac output monitor (EV1000; Edwards Lifesciences, Irvine, USA) was used to measure cardiac output (CO), systemic vascular resistance (SVR), cardiac index (CI), and stroke volume variation (SVV) (Figure 2).

**Figure 3:**
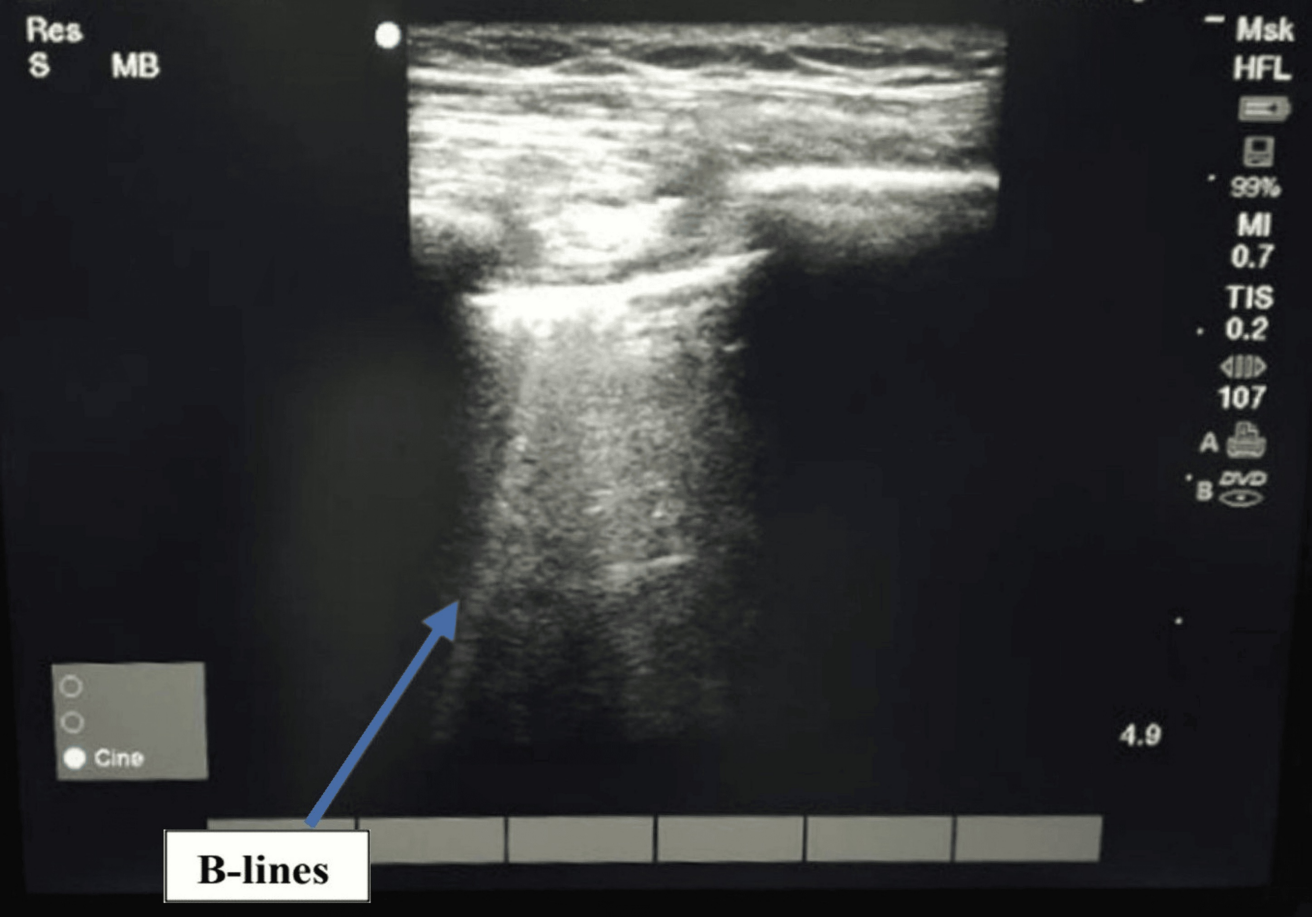
Lung USG showing B-lines Lung USG of the right basal segment showing occasional B-lines, which are hyperechoic vertical lines extending from the pleural line towards the bottom of the screen. Presence of more than three lines is indicative of excessive extravascular lung water.

After adequate padding, the patient was positioned in the right lateral position, and robotic arms were docked (SSi Mantra Surgical Robotic System; SS Innovations International Inc., India). The surgery lasted 4.5 hours with a blood loss of 500 mL, requiring one packed red blood cell (PRBC) transfusion over five hours. ABG analysis showed a pH of 7.2, PaO_2_ of 146 mmHg, PaCO_2_ of 45 mmHg, and bicarbonate level of 20 mmol/L, prompting the start of sodium bicarbonate infusion. Four-quadrant chest ultrasound was performed to check for pulmonary congestion at various time points (before docking, after surgery, and during the postoperative period) (Figure 3).

**Figure 4:**
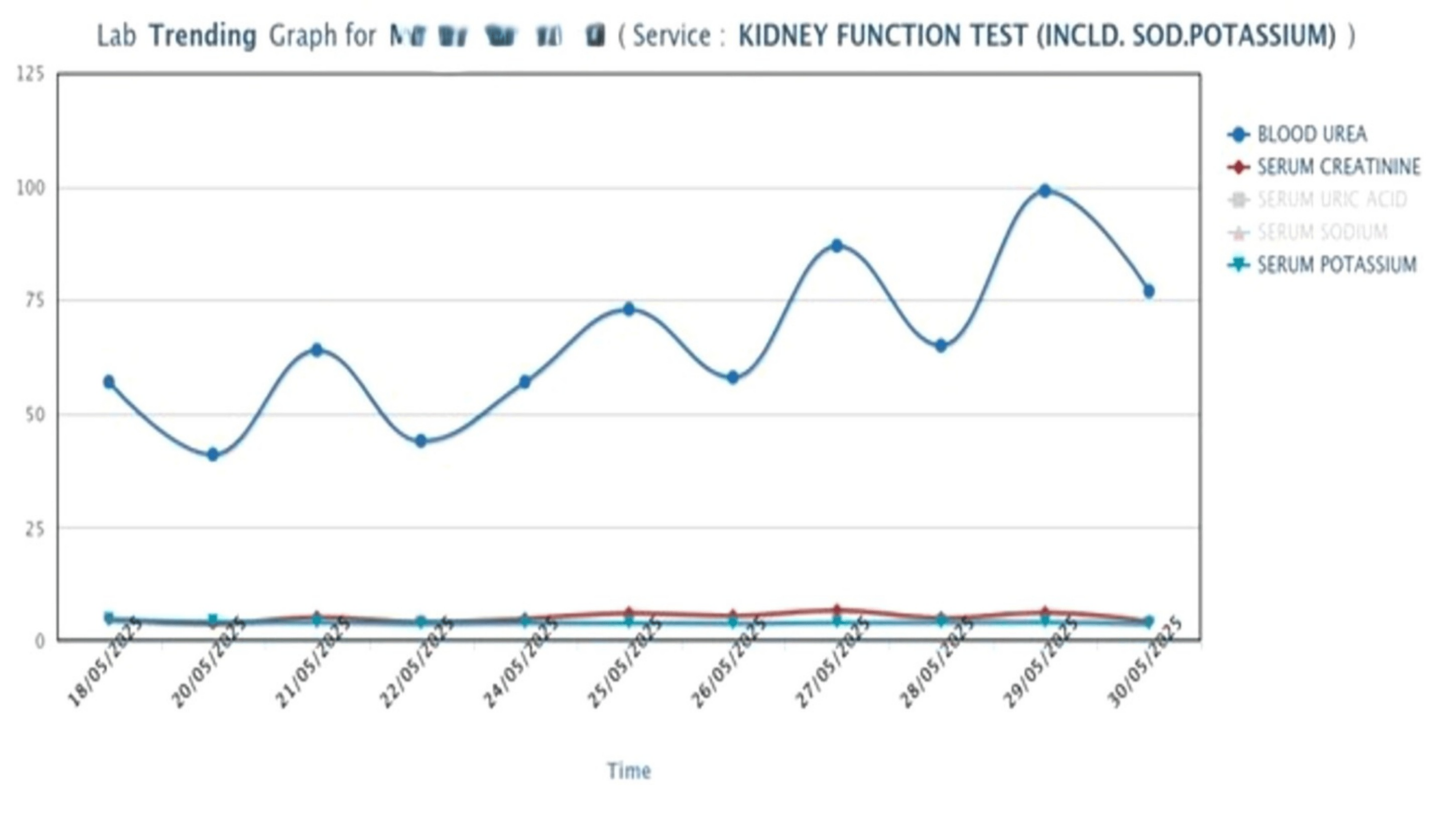
Graph showing trends in KFT KFT: Kidney function test

After the completion of surgery, a 5-6 cm incision was made to remove the specimen, and a USG-guided unilateral transversus abdominis plane (TAP) block with 10 mL of 0.125% bupivacaine was administered. The patient was reversed with neostigmine and glycopyrrolate and shifted to the surgical intensive care unit (SICU), where he was conscious, oriented, and pain-free with a visual analog scale (VAS) score of 0. He received paracetamol and tramadol for pain management and was started on intravenous fluid at 30-50 mL/kg. On postoperative day (POD) 1, urea was 57 mg/dL and creatinine was 4.9 mg/L; on POD 2, urea was 72 mg/dL and creatinine was 6.2 mg/L. Electrolytes remained normal. Hemodialysis was performed on POD 2. He was referred to a nephrologist for further management while awaiting a transplant, as guidelines suggest a waiting time of 2-5 years for symptomatic RCC patients (Figure 4).

## Discussion

Robotic-assisted surgeries offer a remarkable advantage for patients reliant on hemodialysis. With their unparalleled precision, reduced operation time, and minimally invasive nature, this advanced technology not only enhances the surgical experience but also paves the way for faster recovery and better patient outcomes. However, caution must be exhibited during the induction of anesthesia, pneumoperitoneum, and in case of excessive blood loss, as these patients may be hypovolemic and can have episodes of severe hypotension.

Current guidelines for patients with end-stage renal disease (ESRD) or anephric dialysis-dependent patients recommend performing dialysis the day before major surgery, managing hypertension with medication or ultrafiltration, and ensuring serum potassium levels are below 5.5 mEq/L on the day of surgery. Hemodialysis prior to surgery helps in achieving a "dry weight," which prevents complications like pulmonary edema and poor wound healing caused by hypervolemia [6]. However, excessive ultrafiltration can lead to hypovolemia and hypotension, especially during anesthesia, blood loss, and the postoperative period. Thus, maintaining an equilibrated, euvolemic state is ideal. Relative blood volume (RBV) change and refilling rate capacity during dialysis can be assessed using tools such as online blood volume sensors [7]. These sensors can assist in monitoring individual patient volume status for better hemodynamic management during hemodialysis [7]. 

More recently, it has also been proposed to extend the use of lung ultrasound in hemodialysis patients for tracking silent fluid accumulation in the lung interstitium (extravascular edema). Ultrasound scanning of the hemi-thorax, from the second to the fourth (on the right side to the fifth) intercostal spaces and from the parasternal to the axillary line, helps in detecting B-lines, which are the signs of interstitial edema and excessive extravascular lung water [8]. USG can detect lung edema even in its early asymptomatic stage. Patients with B-line scores of 15-30 are classified as having moderate lung congestion, and those with scores greater than 30 are classified as having severe lung congestion. Our patient at all time points had a B-line score of <5, suggesting no fluid overload. Cardiac and vascular biomarkers, such as atrial natriuretic peptide (ANP), brain natriuretic peptide (BNP), and N-terminal pro-B-type natriuretic peptide (NT-proBNP), have also been used extensively for assessing fluid overload. In our patient, NT-proBNP was 1500 pg/mL despite no volume overload or cardiac decompensation. The clearance of NT-proBNP is primarily renal; therefore, a low GFR may be the reason for the increased NT-proBNP in our patient [9].

The intricate pathophysiological changes occurring in patients with CKD arise from a complex interplay of heightened circulating inflammatory mediators, a state of hypercoagulability, arterial calcification, and endothelial dysfunction. Those with grade 4/5 CKD often find themselves in a challenging battle against fluid overload. As glomerular filtration steadily declines, sodium and water are retained, leading to an increase in hydrostatic pressure. This pressure drives fluid into the extravascular space, manifesting itself with symptoms of generalized and pulmonary edema. Consequently, maintaining fluid balance becomes a delicate yet crucial task. This tendency towards the development of fluid overload can not only cause pulmonary edema and pleural effusions but also result in a decrease in pulmonary compliance, a reduction in functional residual capacity, and an increase in ventilation-perfusion mismatch.

Like our patient, most patients on hemodialysis often experience free sodium accumulation, leading to left ventricular hypertrophy, vascular stiffness, and hypertension [10,11]. Left ventricular hypertrophy can occur in these patients due to chronic volume and pressure overload. Volume overload is typically caused by water and sodium retention, the presence of an arteriovenous fistula, or chronic anemia, which leads to a hyperdynamic circulation characterized by increased stroke volume and tachycardia. On the other hand, pressure overload is primarily caused by hypertension and arteriosclerosis. Hypertrophy of the left ventricle is associated with myocardial fibrosis and impaired myocardial relaxation, which can lead to diastolic dysfunction and arrhythmias. Furthermore, tissue sodium accumulation may contribute to metabolic and inflammatory disorders (e.g., insulin resistance, protein-energy wasting) that increase cardiovascular risk [12,13].

These patients are also prone to hyperkalemia due to distal renal tubular dysfunction, transcellular potassium shifts exacerbated by factors like hyperglycemia, insulin deficiency, aldosterone resistance, beta-blockers, and acidemia. The use of intravenous beta-adrenergic receptor blockers for the acute management of hypertension can also cause acute hyperkalemia. The perioperative blood glucose goal in patients dependent on hemodialysis is typically between 5 and 10 mmol/L [12]. We had started a dextrose drip in our patient. Hourly blood glucose levels were measured, and the insulin infusion was titrated accordingly.

Metabolic acidosis is a common complication of advanced CKD due to a combination of increased production of non-volatile acids, increased bicarbonate loss (and therefore decreased buffering capacity), decreased renal excretion of acid, and decreased capacity for further respiratory compensation. Acidemia can also cause hyperkalemia. Alkali supplementation should therefore be provided to maintain the serum bicarbonate concentration at a value greater than 18-20 mmol/L. As the pH of the blood in our patient during robotic surgery was 7.2, sodium bicarbonate infusion was started. Thus, serial ABG analysis must be done to detect and treat acidosis. Chronic metabolic acidosis can also lead to osteopenia in these patients; thus, positioning during surgery must be done with care. 

The decreased functioning renal mass also leads to reduced erythropoietin production, abnormal iron metabolism, and reduced red blood cell survival. Erythropoiesis-stimulating agents should be considered when hemoglobin is below 10 g/dL, with iron saturation at least 20% to 30% and ferritin greater than 200 ng/mL [12]. The target hemoglobin concentration should be 10-11.5 g/dL.

Platelet dysfunction and thromboasthenia due to uremia result in decreased platelet adhesion and increased fragility of blood vessel walls, contributing to bleeding disorders. Reduction in adenosine diphosphate (ADP) content, poor aggregation in response to ADP, excessive production of endogenous nitric oxide, decreased levels of thromboxane A2, and improper interaction between von Willebrand factor and platelet glycoprotein IIb-IIIa receptors often result in impaired platelet function. However, prothrombin time and activated partial thromboplastin time may be normal. Thus, platelet mapping or thromboelastography must be done to prevent excessive bleeding during surgery [13].

Paradoxically, reduced fibrinolysis, increased initial fibrin formation, fibrin-platelet interaction, and altered platelet function can also cause a prothrombotic state. The incidence of venous thromboembolism (VTE) increases with decreasing estimated glomerular filtration rate (eGFR), advancing age, and other factors, including immobility and surgery [13].

Goal-directed fluid therapy, utilizing appropriate crystalloids and vasopressors, is crucial, targeting a mean arterial pressure (MAP) of at least 65 mmHg [12]. In case of excessive blood loss, blood and blood product transfusions are necessary. However, close monitoring of volume status and blood chemistry is essential as the ESRD patients are at a higher risk for volume overload, hyperkalemia, and thrombosis after transfusion.

Lastly, an appropriate combination of anesthetic agents with minimal renal excretion and a shorter duration of action is crucial. Fentanyl, paracetamol, dexmedetomidine, and regional blocks provide adequate analgesia [12,13]. However, the use of neuraxial blockade is controversial in dialysis-dependent grade 4/5 CKD patients due to concerns about platelet dysfunction and the risk of epidural hematoma [14]. Dexmedetomidine, in addition to providing analgesia and sedation, also has renoprotective action, and dose alteration is not recommended in CKD patients [15].

However, morphine with an active metabolite, morphine-6-glucuronide, which is eliminated by the kidney, must be avoided in patients with CKD. Non-steroidal anti-inflammatory drugs (NSAIDs) reduce renal blood flow and GFR and can cause acute interstitial nephritis and platelet dysfunction. NSAIDs can also contribute to hyperkalemia. Thus, they should be avoided in CKD patients [13].

Cisatracurium and atracurium are recommended as ideal muscle relaxants in patients with renal disease due to their extra renal metabolism. Some experts recommend rocuronium for rapid sequence induction due to possible delayed gastric emptying in renal dysfunction. However, once rocuronium is bound by sugammadex, it forms a complex that must be excreted renally, and in patients with CKD, this can lead to delayed clearance and prolonged drug action [16].

## Conclusions

Robotic surgeries present a remarkable option for hemodialysis-dependent patients, thanks to their minimally invasive approach. These advanced procedures significantly reduce blood loss and the need for transfusions, while also alleviating pain, shortening hospital stays, and accelerating recovery compared to traditional open surgeries.

For patients lacking kidney function, the risks of hypervolemia, electrolyte imbalances, and acidosis are heightened. Diligent monitoring of perioperative volume status, blood chemistry, and platelet function, as well as performing hemodialysis before and after surgery, are the cornerstones in the management of these patients. Finally, a comprehensive anesthetic plan including regional blocks and non-nephrotoxic drugs not only enhances patient safety but also fosters a more favorable recovery journey.
